# GABAergic Modulation in Movement Related Oscillatory Activity: A Review of the Effect Pharmacologically and with Aging

**DOI:** 10.5334/tohm.655

**Published:** 2021-11-10

**Authors:** Christopher L. Groth, Arun Singh, Qiang Zhang, Brian D. Berman, Nandakumar S. Narayanan

**Affiliations:** 1 Department of Neurology, University of Iowa, Iowa City, IA 52242, US; 2 Division of Basic Biomedical Sciences, University of South Dakota, Vermillion, SD 57069, US; 3 Department of Neurology, Virginia Commonwealth University, Richmond, VA 23298, US

**Keywords:** Gamma aminobutyric acid (GABA), electroencephalography (EEG), event-related desynchronization (ERD), event-related synchronization (ERS)

## Abstract

Gamma-aminobutyric acid (GABA) is a ubiquitous inhibitory neurotransmitter critical to the control of movement both cortically and subcortically. Modulation of GABA can alter the characteristic rest as well as movement-related oscillatory activity in the alpha (8–12 Hz), beta (13–30 Hz, and gamma (60–90 Hz) frequencies, but the specific mechanisms by which GABAergic modulation can modify these well-described changes remains unclear. Through pharmacologic GABAergic modulation and evaluation across the age spectrum, the contributions of GABA to these characteristic oscillatory activities are beginning to be understood. Here, we review how baseline GABA signaling plays a key role in motor networks and in cortical oscillations detected by scalp electroencephalography and magnetoencephalography. We also discuss the data showing specific alterations to baseline movement related oscillatory changes from pharmacologic intervention on GABAergic tone as well as with healthy aging. These data provide greater insight into the physiology of movement and may help improve future development of novel therapeutics for patients who suffer from movement disorders.

## 1. Introduction

Gamma-aminobutyric acid (GABA), is a critically important inhibitory neurotransmitter within the central nervous system shown to impact cortical electrographic activity [1]. A growing body of data in animals and humans supports its critical importance in movement generation and the characteristic oscillatory changes detected on scalp electroencephalography (EEG) during movement [1,2,3]. Furthermore, modulating GABA in hyperkinetic movement disorders, such as dystonia and essential tremor, can improve physical symptoms as well as mitigate abnormal movement-related oscillatory changes detected on EEG and subcortical microelectrodes [1,3,4,5,6,7]. Thus, investigating the GABAergic effect on movement-related oscillatory changes is critical to understanding the functional anatomy of normal and disordered movements with the goal of developing more targeted and better tolerated treatments.

Since first being described by Gastaut et al. in 1952, scalp EEG has been used to capture the movement related potentials of synchronized activity involving large regions of the cortex [8]. Later, in 1972, magnetic brain activity measurements were first detected and became the foundation for the development of magnetoencephalography (MEG) [9]. Through advances in EEG and MEG technology [10], the anatomic location of these movement-related potentials has been better localized and characterized as to the frequency and power shifts from baseline [10,11,12]. These alterations have been categorized as *event-related desynchronizations* (ERD) [13] and *event-related synchronizations* (ERS) [14]. Although these oscillatory changes are well described, the underlying mechanisms of these changes remain unclear.

This review is divided into four main parts. First, in section one, we summarize the overall importance of GABA for motor control and review normal movement-related oscillatory changes measured with scalp EEG and MEG. Next, in section two and three, we provide an overview of the currently known alterations to these oscillatory changes seen using GABAergic modulation with pharmacologic interventions, as well as normal aging. In the discussion, we provide an overview of how GABA may ultimately affect movement-related oscillations and therefore, movement. Finally, we discuss potential future explorations to better understand GABAergic contributions to movement and how this may lead to the development of novel treatments for movement disorders.

### 1.1. GABA and physiologic networks

GABA is a neurotransmitter that is ubiquitous among inhibitory neurons throughout the brain [15]. GABA binds to two main classes of receptors, GABA_A_ and GABA_B_. Ionotropic ligand-gated GABA_A_ receptors mediate fast synaptic inhibition by allowing an influx of chloride ions that hyperpolarize neurons. They represent the majority of GABAergic receptors in the brain, and, given the diverse receptor subunits and potential isoform combinations, they have the most pharmacologic binding sites. Metabotropic GABA_B_ receptors mediate slow, prolonged inhibitory signaling through G-coupled proteins [16]. These two receptor classes are distributed across specific brain regions in different isoform combinations, and contributing to the unique synaptic activity of each brain region [17].

In the context of motor control, there are three major network elements by which GABA might exert inhibitory action. The first is through interneurons in motor and premotor cortex. Interneurons are markedly diverse in their connections and signaling, but are all primarily inhibitory and GABAergic in nature [18]. They can project locally and powerfully inhibit other interneurons in Layer II/III or pyramidal cells in Layer V, including those that project subcortically to the basal ganglia, pons, or directly to spinal cord motor neurons via the corticospinal tract [19].

In rat studies, characteristic beta oscillations were noted to arise from the pyramidal cells of Layer II and V with frequency modulation resulting from GABAergic interneurons providing phasic GABA mediated inhibition at beta frequencies [20]. Furthermore, in recordings at the primary motor cortex, beta and gamma oscillations were abolished with the GABA_A_ antagonists picrotoxin and gabazine [21,22]. Additionally, study of pyramidal cells from Layer V of rats have also confirmed that GABA agonist administration was sufficient to yield the characteristic beta power increase seen on standard scalp EEG at the cellular level [23].

The second network is in the basal ganglia, where medium spiny neurons integrate input from diverse cortical and subcortical sources, sending progressively convergent, inhibitory GABAergic projections to subcortical and thalamic targets [24,25]. Microinjection of GABAergic medications into the globus pallidus has been shown to alter inhibitory postsynaptic current as well as cause alterations in rodent behavior with resultant turning that can be rescued with concomitant administration of flumazenil [26,27].

The third is in the cerebellum, which uses GABA as a major transmitter in Purkinje cells [28] and prominently affects movement [29]. Modulation of cellular and movement-related activity with GABAergic manipulation has also been confirmed in cerebellar slices and animal models, respectively [30,31]. Thus, GABA is quite obviously integral for the development of cortical oscillatory rhythms and its modulation is sufficient to affect beta frequency oscillations in *in vitro* studies.

### 1.2 Baseline movement-related oscillatory changes

ERD is characterized by a decrease in the power of alpha (8–12 Hz) and beta (13–30 Hz) frequencies in the primary motor cortex prior to and during movement [32]. This decrease in power can be detected up to two seconds before the initiation of movement and persists through the movement [11]. The onset of ERD is first detected contralateral to the side of movement, but then is detected bilaterally at movement onset [32]. Alpha and beta-frequency changes are temporally and spatially independent. The beta event related desynchronization (beta-ERD) occurs prior to and ends before the alpha event related desynchronization [33]. The beta frequency is detected at the precentral (motor) cortex, whereas the alpha frequency is detected at the postcentral (somatosensory) cortex [34]. Beta frequency modulation of movement is not limited to the motor cortex, though. Connections between the motor cortex and the supplementary motor cortex, inferior frontal gyrus, and parietal cortex are shown to affect accuracy of movement and be modulated with the beta band frequency [35,36].

The alpha-ERD is thought to correlate with decreased inhibition for sensorimotor modulation of task-specific information [37]. This information then assists with the planning phase of movement [38]. The beta-ERD is thought to arise more from the thalamo-cortical networks that specifically activate motor areas of the cortex by reducing excitability thresholds [11]. Furthermore, the beta-ERD is suspected to contribute to movement through contributing information related to the duration, speed, and type of movement to be generated [39]. Given the evidence of GABAergic contribution to beta frequency generation in cortical interneurons [20,21,22,23], the generation of ERDs appear inherently tied to the inhibitory drive of GABAergic interneurons that project to interneurons in Layer II/III or pyramidal cells in Layer V [19].

ERS occurs in the primary motor cortex at two time points within the movement which are associated with two different frequencies: gamma (60–90 Hz) [40] at the initiation of movement and beta at the termination of movement [12]. The first ERS at gamma frequency occurs at, or immediately prior to, the initiation of movement with a short lasting (<1 s) burst of gamma frequency synchronization. This movement-related gamma synchronization (MRGS) occurs in the sensorimotor cortex contralateral to the side of movement [12]. The second ERS occurs in the bilateral precentral (motor) cortex at the cessation of movement in the beta frequency range (beta-ERS). This event raises the beta frequency power above baseline for roughly 1 second [11,41]. Both beta and gamma power returns to baseline before the next movement is initiated if movements are not continuously repeated [12].

MRGS is considered to be more associated with planning or initiation of movement than with its continuation [42,43]. Furthermore, excessive phase-amplitude-coupling (PAC) of alpha/beta frequencies with gamma frequencies in the motor cortex has been observed in hypokinetic disorders, such as Parkinson’s disease, and is believed to be one mechanism for the observed movement deficits in Parkinson’s disease [44,45]. Whereas, beta-ERS has been suggested to represent an inhibition of the motor cortex [46] or somatosensory reafference [47] for which there is debate as to the importance of GABAergic mediation [1,3,7].

## 2. Pharmacologic GABAergic modulation of cortical potentials

Given significant evidence that GABA is integral to the control of neuronal network oscillations within the beta frequency range of the motor cortex, most early studies of GABAergic contributions to movement-related oscillatory changes were conducted using pharmacologic evaluation [1]. From this work, the use of GABAergic medications, as well as GABA levels, have been shown to alter both movement-related and rest oscillatory changes within the primary motor cortex [1,3,7,48,49].

### 2.1 Event-related desynchronizations

GABAergic medications, such as benzodiazepines (GABA_A_ positive allosteric modulator) and tiagabine (GABA reuptake inhibitor), alter movement-related beta-ERD significantly [1,7]. Administration of diazepam increases beta power at rest which leads to a larger total decrease in power during ERD than is seen when the onset of beta-ERD starts at baseline without GABAergic medications [7]. Thus, the rate of desynchronization increases to accommodate the drop in beta power that is required for movement [1]. Moreover, the beta peak frequency (frequency with the largest observed power) is decreased after administration of diazepam [1]. Of note, the effect of *in vivo* GABA levels on ERD as measured by magnetic resonance spectroscopy (MRS), has not been reported. For alpha frequencies, administration of diazepam was not noted to lead to significant changes to power until higher doses of diazepam were used. Even at that point, however, power was only slightly reduced [50].

### 2.2 Event-related synchronizations

With movement, there are two ERS events: MRGS and beta-ERS. MRGS occurs first, roughly at movement onset, but neither MRGS power nor frequency are altered by pharmacologic modulation of the GABAergic system [1,7]. However, GABA levels measured by MRS in the sensorimotor cortex do positively correlate with MRGS peak frequency, though no correlation is seen with power [3].

The second ERS event occurring at the cessation of movement, is the (beta-ERS). Pharmacologic GABAergic modulation can affect beta-ERS, but the results have been mixed. With administration of diazepam, there is an additional increase in beta-ERS power from baseline, but does not show any significant change in residual power with respect to spontaneous beta power [1]. However, beta-ERS power has less increase from baseline after administration of tiagabine [7]. With either drug, though, the beta-ERS peak frequency decreases compared to placebo [1,7]. When measuring with MRS, baseline GABA levels in the sensorimotor region were positively correlated with beta-ERS power [3,51], but no correlation was found with beta-ERS peak frequency [51].

### 2.3 Cortical rest frequencies

Benzodiazepine administration increases resting beta frequency power but decreases beta frequency peak power [1]. Additionally, beta peak frequency is positively correlated with GABA levels in the sensorimotor cortex contralateral to the side of movement, but not ipsilateral, as measured by MRS [49]. Administration of zolpidem (GABA_A_ positive allosteric modulator) has been shown to reduce alpha power, diazepam has had more mixed results in effect on alpha power, and tiagabine has been noted to have no significant change on alpha frequency power [48,52,53,54].

Gamma frequency oscillations during rest are not affected by GABAergic modulation with benzodiazepines [1]. Further investigation of the effects of GABA levels on rest gamma frequency peak power and peak frequency power at the sensorimotor cortex in the literature is lacking. However, through analysis of the visual cortex, GABA levels as measured by MRS have been shown to positively correlate with rest gamma frequency, but not rest gamma power [55].

### 2.4 GABAergic medications summary

These above-mentioned medications, such as benzodiazepines, increase GABAergic tone, increase overall inhibition, and modulate overall movement [1,48,49]. As such, these medications are frequently used for therapeutic interventions in hyperkinetic movement disorders, such as dystonia and essential tremor, that are demonstrated to have abnormalities in inhibitory signaling [4,56,57,58,59,60,61], but also in Parkinson disease, a movement disorder with significant hypokinesis [62]. Thus, modulating GABA and overall inhibitory signaling is vital to the treatment of movement disorders, but improved understanding of these medications is critical given that our current GABAergic medications have frequent side effects of sedation, increased fall risk, and potential for abuse [63,64].

Zolpidem has been shown to increase rest beta frequencies but decrease alpha frequencies [52]. However, tiagabine showed increases in power for delta through beta frequencies, but the increases in beta and alpha frequencies were absent to reversed in the parietal/occipital regions [52]. Similar medication-specific effects have been seen in transcranial magnetic simulation (TMS)-evoked EEG changes between diazepam, alprazolam, zolpidem, and baclofen [65]. These effects may even be dose dependent as low dose zolpidem appears to increase GABA_A_ receptors in a kinetically slow desensitized state while higher doses favor rapid transitions into and out of desensitized states [23].

Within patients with Parkinson disease, this difference in effect may manifest clinically as administration of low dose zolpidem improved speed of movement compared to higher doses that appeared to worsen it [5]. Furthermore, baseline GABA levels may ultimately affect the pharmacologic or neuromodulatory effect of these medications [66]. Thus, further exploration of the pharmacological and pharmacokinetic effect of GABAergic medications is needed to better understand their effect and design better medications with less significant side effects.

## 3. Effects of aging on modulation of cortical potentials

In addition to the above-noted effects of pharmacologic GABAergic modulation on movement-specific and rest oscillatory changes, advancement in age has also been shown to alter the cortical oscillatory patterns of neurons within the primary motor cortex region [39,67,68,69,70]. With aging, there is an apparent decrease in GABA levels in various regions throughout the brain [70,71,72,73,74]. However, it is not clear that these GABA changes are sufficient to account for all movement-related oscillatory changes.

### 3.1 Event-related desynchronizations

With increasing age, beta-ERD is noted to be greater (larger decrease in power from baseline) comparing younger healthy adults to older healthy adults, and linearly changes over time [39,68]. The etiology of this increased ERD is due to an elevated beta frequency power at rest. [39]. When comparing beta-ERD in adults and children, it has been noted that adults have onset of beta-ERD as early as two seconds prior to the onset of movement, leading to a longer overall duration of beta-ERD, whereas children have onset of beta-ERD when movement is initiated [69]. Furthermore, children have smaller amplitude beta-ERD compared to adults, though older children (ages 11–13) have a larger amplitude beta-ERD than younger children (ages 4–6) [69,75]. Reporting on laterality of this finding has been mixed with findings being reported both as only unilateral to the movement [68] and bilateral [39].

### 3.2 Event-related synchronizations

MRGS is detected in children as young as four years old and remains present throughout life. However, among healthy adults, the peak frequency decreases with advancing age [3,69]. Interestingly, MRGS power has been shown to be greatest in 11–13 year old subjects compared to 4–6 year old and adult subjects, though the etiology of this difference is unclear [69]. Across the age spectrum, adults have higher beta-ERS power compared to children. Additionally, beta-ERS power appears to increase with age (ages 4–6 vs ages 11–13 vs adults), although reliable detection of beta-ERS power in young children (ages 4–6) has been difficult [69].

### 3.3 Cortical rest frequencies

Within the beta frequency range, there is an increased power at rest [39,68] and a reported non-significant trend (p = 0.06) toward a lower peak beta-frequency associated with aging in adults [68]. Rest gamma frequency power has in contrast been found to increase with age [67]. It is not clear whether the gamma peak frequency changes with healthy aging, but it has been reported at least in one study that peak frequency and power are not correlated with each other [55].

### 3.4 Aging summary

With healthy aging there appears to be a general decrease in inhibitory tone [71,72,73,76] noted by alterations in movement related synchronizations, decreased GABA levels [70,71,72,73,74], and reduced inhibition by measuring short-interval intracortical inhibition (SICI) [73,74,77]. These noted alterations in inhibitory signaling are potentially clinically significant. Gamma oscillation transcranial alternating current stimulation (gamma-tACS) is known to modulate and enhance GABAergic neurotransmission. However, within older adults (65.3+/– 9.5 years old), this modulation is reduced and negatively correlated with age [77]. Further, subjects with less increase in GABA inhibition from gamma-tACS show slower short-term learning [78]. This inhibitory deficit has been also noted to impact the speed of reaction in older adults [72]. Enhancing the GABAergic neuron in the hippocampus of a mouse model Alzheimer’s disease also showed improvement in cognitive tasks as well as baseline cortical rhythms [79]. As such, reduced inhibitory signaling within the aging brain has significant clinical impact and merits further study, but the full spectrum of movement associated changes seen on EEG are likely a result of a more complicated aging process involving neurotransmitter imbalance, as a purely GABAergic explanation is unable to account for the discrepancies in movement-related oscillatory changes seen between young and old [80]. Thus, further work is needed to better understand the contributions and changes from other important neurotransmitters such as serotonin, dopamine, norepinephrine, etc.

## 4. Discussion

Baseline movement-related oscillations have characteristic alterations related to both pharmacologic GABAergic modulation and aging (***Table 1***). Interestingly, the reported oscillatory activity during movement is similar for these two states despite some possible differences in the inhibitory state of the two conditions. Intrinsic GABAergic modulation is critical for the control of movement as measured through electrophysiologic recordings of the cortex, but the understanding of the impact of pharmacologic manipulation and aging on the underlying pathophysiology remains unclear.

**Table 1:** Cortical power and peak frequencies as a function of pharmacologic modulation and age.


ELECTROGRAPHIC ACTIVITY		GABAERGIC PHARMACOLOGIC MODULATION	AGING

Beta Frequency (13–30 Hz) Event Related Desynchronization	*Power*	↑	↑

	*Peak Frequency*	↓	─

Beta Frequency (13–30 Hz) Event Related Synchronization	*Power*	↔^D^ ↓^T^	↑

	*Peak Frequency*	↓	─

Gamma Frequency (60–150 Hz) Event Related Synchronization	*Power*	↔	─

	*Peak Frequency*	↔	↓

Rest Cortical Beta Frequency	*Power*	↑	↑

	*Peak Frequency*	↓	↓*

Rest Cortical Gamma Frequency	*Power*	↔	↑

	Peak Frequency	↔	─


Legend: ↑ (increased), ↓ (decreased), ↔ (unaltered), ─ (unreported), * (not statistically significant), ^D^ (diazepam), ^T^ (tiagabine).

### 4.1 GABA effect on electrophysiologic measures

The beta-ERD is strongly linked to GABAergic inhibition and reflects the amount of inhibitory signaling [19]. Thus, with increased inhibition from GABAergic medications that increase beta frequencies, there is a strongly increased beta-ERD [7]. As such, an increased beta-ERD would be expected to be associated with impaired movement given the extra amount of desynchronization required for movement initiation. In subjects who have taken benzodiazepines [81] and older individuals there was a larger beta-ERD that was associated with increased duration of movements [39]. However, older adults are also noted to have decreased overall inhibitory signaling on neurochemical as well as electrophysiologic measures as opposed to increased inhibition [71,72]. Thus, an increased beta-ERD seems to be consistently associated with impaired movement, but the factors contributing to an abnormal beta-ERD can be multifactorial and not simply related to GABAergic alterations.

For the alpha-ERD, GABAergic manipulation pharmacologically has not been shown to have significant effect. Thus, a leading hypothesis has been that alpha-ERD is not primarily a GABAergic process [50,54]. This is further supported by evidence of it representing an excitatory state that promotes targeted movement [37,82]. With training of a given movement, however, the alpha-ERD is noted to decrease, especially in athletes, as less overall cortical activation is needed to yield the desired movements [83]. In older individuals vs younger individuals, a larger alpha-ERD is noted to correlate with slower movements [84]. As such, the slower speed of movements seen in older individuals may not be directly related to an underlying inhibitory/GABAergic process as suggested in the combined evidence with the beta-ERD.

The overall GABAergic dependence of MRGS is not well established. Within the visual system, peak frequency of MRGS is well described to be positively correlated with GABA levels, but this is less clear in the sensorimotor cortex [85]. Peak gamma frequency appears to decrease with age [3], while GABA is decreasing [70,71,72,73,74]. However, modulation with tiagabine and diazepam does not appear to affect MRGS [1,7] nor was it linked to GABA levels measured by MRS [3]. Nevertheless, MRGS likely plays a role in movement as the peak frequency of MRGS may be dependent upon the motion produced as it assists with motor initiation [85].

Finally, the beta-ERS as noted above, does not have a proven direct connection with the GABAergic system [1,3,7]. However, there is strong support for beta-ERS to represent active inhibition of motor cortex related to processes over distributed networks [47,86]. As such, the beta-ERS may represent more of top-down inhibitory control through a more widely integrated sensorimotor network. Overall parameters of beta-ERS including the amplitude and time-to-peak of beta-ERS can be modulated via altered task duration, force output, and rate of force development [87,88].

### 4.4 Future Explorations

One significant limitation of current studies of the effects of GABAergic modulation and aging on movement related oscillatory changes is the inability to evaluate electrographic activity outside of the cerebral cortex. GABAergic signaling is critical to movement control in the basal ganglia and the cerebellum, both of which modulate the cortical signal [29,89,90]. Given their limited anatomic accessibility, though, the basal ganglia and cerebellum has been largely unexplored in normal, healthy human controls. However, dysfunction in these key areas of the motor network is linked to several movement disorders including Parkinson disease, dystonia, and essential tremor and have known importance in GABA associated movement generation [91,92,93]. These movement disorders are known to have altered movement-related oscillatory changes and can be symptomatically improved with pharmacologic modulation of GABA [1,3,4,5,6,7,94]. As such, they represent an opportunity for deeper investigation of movement control.

A key opportunity for improved exploration of GABAergic modulation of subcortical structures is through deep brain stimulation (DBS). As part of the treatment for several movement disorder, DBS is used to target structures including the subthalamic nucleus (STN), globus pallidus internus (GPi), and thalamus. The electrode placement allows opportunity to both record as well as then modulate activity at these structures. As an example, animal models have shown that modulation of the GPi is sufficient to induce beta frequency oscillations in the cortex [95]. Furthermore, in human movement disorders, DBS of the GPi increases GABAergic inhibition within the GPi leading to a decrease in thalamocortical activity, improved pathologic cortical activity, and disease symptomatology of both dystonia and Parkinson disease [5,6,90,96,97,98,99].

The cerebellum, in contrast to subcortical structures that are only accessible through medical treatments of a select number of movement disorders, has a more accessible location. As such, with advanced imaging capabilities, it has begun to be studied for its role in movement generation in normal, healthy controls. MEG imaging in children and adolescents of the cerebellum has shown a movement-related beta-ERD as well as beta-ERS that coincides with the observed oscillatory changes in the cortex [100]. Thus, the cerebellum provides a unique structure for potential exploration and development of therapeutic interventions for movement disorders.

## 5. Conclusion

In summary, both pharmacological modulation of the GABAergic system and aging have clear and identifiable consequences on movement-related oscillatory changes. However, the full etiology of these changes remains unclear. These two states yield similar electrophysiologic findings despite the apparently disparate GABAergic alterations. However, as there is a significant lack of reported findings related to GABAergic modulation on alpha-frequency ERD and MRGS, these perceived similarities may only exist for beta-frequency movement-related oscillatory changes. Furthermore, the underlying mechanisms for generation of the beta-ERD in aging may be more complex and involve a neurotransmitter imbalance associated with aging.

Future studies will be needed to specifically address the correlation between movement-related oscillatory changes across all frequencies and GABA levels within the motor network (i.e., motor cortex, thalamus, basal ganglia, and cerebellum) in response to pharmacologic management and aging. Such studies will help disambiguate the overall effect GABA levels have on oscillatory changes. Furthermore, conceptual understanding of the pharmacologic effects from GABAergic medications, such as benzodiazepines, will help to advance future therapeutic developments. GABAergic medications are frequently used to treat a myriad of neurologic disorders, but they are also frequently associated with significant side effect of sedation, increased fall risk, and potential for abuse that limit their current usage and overall safety [63,64]. Development of targeted, channel specific medications may very well lead to significant improvements in overall disease management.

Additionally, a greater understanding of how the basal ganglia and cerebellum impact event-related synchronizations/desynchronizations through GABAergic changes remains an area that needs further research. Both structures have significant GABAergic involvement, as well as clearly evident impacts on movement and cortical movement-related oscillatory changes when GABA is modulated. Future exploration of the effects of DBS on cortical and subcortical GABAergic activity in movement disorders provides an exciting opportunity to study network connections. Furthermore, advanced capabilities in EEG and MEG technology is will allow similar insight into cerebellar function, but in a potential healthy control population. As such, an improved overall understanding of these structures, including their electrophysiologic alterations with movement and their connections with the entire motor network, will assist with developing of novel treatments for multiple movement disorders.

## Financial Disclosures of all Authors (for the preceding 12 months)

C.G.: Declaration of interest: none

A.S.: Declaration of interest: none

Q.Z.: Declaration of interest: none

B.B.: Dr. Brian D. Berman in the last 24 months has received research grant support from the Dystonia Coalition (receives the majority of its support through NIH grant NS065701 from the Office of Rare Diseases Research in the National Center for Advancing Translational Science and National Institute of Neurological Disorders and Stroke), Benign Essential Blepharospasm Research Foundation, Tools4Patient, Mary Rossick Kern and Jerome H. Kern, Parkinson’s Foundation, and the VCU School of Medicine. He is on the medical advisory board of the Benign Essential Blepharospasm Research Foundation and the National Spasmodic Torticollis Association.

N.N.: Declaration of interest: none

## References

[1] Hall SD, Stanford IM, Yamawaki N, McAllister CJ, Ronnqvist KC, Woodhall GL, et al. The role of GABAergic modulation in motor function related neuronal network activity. Neuroimage. 2011; 56(3): 1506–10. DOI: 10.1016/j.neuroimage.2011.02.025 21320607

[2] Boecker H. Imaging the role of GABA in movement disorders. Curr Neurol Neurosci Rep. 2013; 13(10): 385. DOI: 10.1007/s11910-013-0385-9 23990356

[3] Gaetz W, Edgar JC, Wang DJ, Roberts TP. Relating MEG measured motor cortical oscillations to resting gamma-aminobutyric acid (GABA) concentration. Neuroimage. 2011; 55(2): 616–21. DOI: 10.1016/j.neuroimage.2010.12.077 21215806 PMC3411117

[4] Elias WJ, Shah BB. Tremor. JAMA. 2014; 311(9): 948–54. DOI: 10.1001/jama.2014.1397 24595779

[5] Prokic EJ, Stanford IM, Woodhall GL, Williams AC, Hall SD. Bradykinesia Is Driven by Cumulative Beta Power During Continuous Movement and Alleviated by Gabaergic Modulation in Parkinson’s Disease. Front Neurol. 2019; 10: 1298. DOI: 10.3389/fneur.2019.01298 31920922 PMC6933612

[6] Amtage F, Feuerstein TJ, Meier S, Prokop T, Piroth T, Pinsker MO. Hypokinesia upon Pallidal Deep Brain Stimulation of Dystonia: Support of a GABAergic Mechanism. Front Neurol. 2013; 4: 198. DOI: 10.3389/fneur.2013.00198 24367353 PMC3851850

[7] Muthukumaraswamy SD, Myers JF, Wilson SJ, Nutt DJ, Lingford-Hughes A, Singh KD, et al. The effects of elevated endogenous GABA levels on movement-related network oscillations. Neuroimage. 2013; 66: 36–41. DOI: 10.1016/j.neuroimage.2012.10.054 23110884

[8] Gastaut H, Terzian H, Gastaut Y. Study of a little electroencephalographic activity: rolandic arched rhythm. Mars Med. 1952; 89(6): 296–310. 12991978

[9] Cohen D. Magnetoencephalography: detection of the brain’s electrical activity with a superconducting magnetometer. Science. 1972; 175(4022): 664–6. DOI: 10.1126/science.175.4022.664 5009769

[10] Pfurtscheller G, Berghold A. Patterns of cortical activation during planning of voluntary movement. Electroencephalogr Clin Neurophysiol. 1989; 72(3): 250–8. DOI: 10.1016/0013-4694(89)90250-2 2465128

[11] Pfurtscheller G, Lopes da Silva FH. Event-related EEG/MEG synchronization and desynchronization: basic principles. Clin Neurophysiol. 1999; 110(11): 1842–57. DOI: 10.1016/S1388-2457(99)00141-8 10576479

[12] Pfurtscheller G, Graimann B, Huggins JE, Levine SP, Schuh LA. Spatiotemporal patterns of beta desynchronization and gamma synchronization in corticographic data during self-paced movement. Clin Neurophysiol. 2003; 114(7): 1226–36. DOI: 10.1016/S1388-2457(03)00067-1 12842719

[13] Pfurtscheller G. Graphical display and statistical evaluation of event-related desynchronization (ERD). Electroencephalogr Clin Neurophysiol. 1977; 43(5): 757–60. DOI: 10.1016/0013-4694(77)90092-X 72657

[14] Pfurtscheller G. Event-related synchronization (ERS): an electrophysiological correlate of cortical areas at rest. Electroencephalogr Clin Neurophysiol. 1992; 83(1): 62–9. DOI: 10.1016/0013-4694(92)90133-3 1376667

[15] Sieghart W, Sperk G. Subunit composition, distribution and function of GABA(A) receptor subtypes. Curr Top Med Chem. 2002; 2(8): 795–816. DOI: 10.2174/1568026023393507 12171572

[16] Chebib M, Johnston GA. The ‘ABC’ of GABA receptors: a brief review. Clin Exp Pharmacol Physiol. 1999; 26(11): 937–40. DOI: 10.1046/j.1440-1681.1999.03151.x 10561820

[17] Solomon VR, Tallapragada VJ, Chebib M, Johnston GAR, Hanrahan JR. GABA allosteric modulators: An overview of recent developments in non-benzodiazepine modulators. Eur J Med Chem. 2019; 171: 434–61. DOI: 10.1016/j.ejmech.2019.03.043 30928713

[18] Tremblay R, Lee S, Rudy B. GABAergic Interneurons in the Neocortex: From Cellular Properties to Circuits. Neuron. 2016; 91(2): 260–92. DOI: 10.1016/j.neuron.2016.06.033 27477017 PMC4980915

[19] Sun Q, Li X, Ren M, Zhao M, Zhong Q, Ren Y, et al. A whole-brain map of long-range inputs to GABAergic interneurons in the mouse medial prefrontal cortex. Nat Neurosci. 2019; 22(8): 1357–70. DOI: 10.1038/s41593-019-0429-9 31285615

[20] Lacey MG, Gooding-Williams G, Prokic EJ, Yamawaki N, Hall SD, Stanford IM, et al. Spike firing and IPSPs in layer V pyramidal neurons during beta oscillations in rat primary motor cortex (M1) in vitro. PLoS One. 2014; 9(1): e85109. DOI: 10.1371/journal.pone.0085109 24465488 PMC3896371

[21] Yamawaki N, Stanford IM, Hall SD, Woodhall GL. Pharmacologically induced and stimulus evoked rhythmic neuronal oscillatory activity in the primary motor cortex in vitro. Neuroscience. 2008; 151(2): 386–95. DOI: 10.1016/j.neuroscience.2007.10.021 18063484

[22] Roopun AK, Middleton SJ, Cunningham MO, LeBeau FE, Bibbig A, Whittington MA, et al. A beta2-frequency (20–30 Hz) oscillation in nonsynaptic networks of somatosensory cortex. Proc Natl Acad Sci U S A. 2006; 103(42): 15646–50. DOI: 10.1073/pnas.0607443103 17030821 PMC1592532

[23] Prokic EJ, Weston C, Yamawaki N, Hall SD, Jones RS, Stanford IM, et al. Cortical oscillatory dynamics and benzodiazepine-site modulation of tonic inhibition in fast spiking interneurons. Neuropharmacology. 2015; 95: 192–205. DOI: 10.1016/j.neuropharm.2015.03.006 25797493

[24] Shink E, Smith Y. Differential synaptic innervation of neurons in the internal and external segments of the globus pallidus by the GABA- and glutamate-containing terminals in the squirrel monkey. J Comp Neurol. 1995; 358(1): 119–41. DOI: 10.1002/cne.903580108 7560274

[25] Smith Y, Wichmann T, DeLong MR. Synaptic innervation of neurones in the internal pallidal segment by the subthalamic nucleus and the external pallidum in monkeys. J Comp Neurol. 1994; 343(2): 297–318. DOI: 10.1002/cne.903430209 8027445

[26] Chen L, Yung WH. Effects of the GABA-uptake inhibitor tiagabine in rat globus pallidus. Exp Brain Res. 2003; 152(2): 263–9. DOI: 10.1007/s00221-003-1549-7 12879169

[27] Chen L, Savio Chan C, Yung WH. Electrophysiological and behavioral effects of zolpidem in rat globus pallidus. Exp Neurol. 2004; 186(2): 212–20. DOI: 10.1016/j.expneurol.2003.11.003 15026257

[28] Hirano T. GABA and Synaptic Transmission in the Cerebellum. Handbook of the Cerebellum and Cerebellar Disorders. 2013; 881–93. DOI: 10.1007/978-94-007-1333-8_36

[29] Bostan AC, Strick PL. The basal ganglia and the cerebellum: nodes in an integrated network. Nat Rev Neurosci. 2018; 19(6): 338–50. DOI: 10.1038/s41583-018-0002-7 29643480 PMC6503669

[30] Kaneda M, Farrant M, Cull-Candy SG. Whole-cell and single-channel currents activated by GABA and glycine in granule cells of the rat cerebellum. J Physiol. 1995; 485(Pt2): 419–35. DOI: 10.1113/jphysiol.1995.sp020739 7545231 PMC1158002

[31] Holdefer RN, Houk JC, Miller LE. Movement-related discharge in the cerebellar nuclei persists after local injections of GABA(A) antagonists. J Neurophysiol. 2005; 93(1): 35–43. DOI: 10.1152/jn.00603.2004 15331620 PMC2590627

[32] van Wijk BC, Beek PJ, Daffertshofer A. Neural synchrony within the motor system: what have we learned so far? Front Hum Neurosci. 2012; 6: 252. DOI: 10.3389/fnhum.2012.00252 22969718 PMC3432872

[33] Erbil N, Ungan P. Changes in the alpha and beta amplitudes of the central EEG during the onset, continuation, and offset of long-duration repetitive hand movements. Brain Res. 2007; 1169: 44–56. DOI: 10.1016/j.brainres.2007.07.014 17689502

[34] Ritter P, Moosmann M, Villringer A. Rolandic alpha and beta EEG rhythms’ strengths are inversely related to fMRI-BOLD signal in primary somatosensory and motor cortex. Hum Brain Mapp. 2009; 30(4): 1168–87. DOI: 10.1002/hbm.20585 18465747 PMC6870597

[35] Picazio S, Veniero D, Ponzo V, Caltagirone C, Gross J, Thut G, et al. Prefrontal control over motor cortex cycles at beta frequency during movement inhibition. Curr Biol. 2014; 24(24): 2940–5. DOI: 10.1016/j.cub.2014.10.043 25484293 PMC4274313

[36] Chung JW, Ofori E, Misra G, Hess CW, Vaillancourt DE. Beta-band activity and connectivity in sensorimotor and parietal cortex are important for accurate motor performance. Neuroimage. 2017; 144(Pt A): 164–73. DOI: 10.1016/j.neuroimage.2016.10.008 27746389 PMC5183516

[37] Pfurtscheller G, Andrew C. Event-Related changes of band power and coherence: methodology and interpretation. J Clin Neurophysiol. 1999; 16(6): 512–9. DOI: 10.1097/00004691-199911000-00003 10600019

[38] Jenson D, Bowers AL, Harkrider AW, Thornton D, Cuellar M, Saltuklaroglu T. Temporal dynamics of sensorimotor integration in speech perception and production: independent component analysis of EEG data. Front Psychol. 2014; 5: 656. DOI: 10.3389/fpsyg.2014.00656 25071633 PMC4091311

[39] Heinrichs-Graham E, Wilson TW. Is an absolute level of cortical beta suppression required for proper movement? Magnetoencephalographic evidence from healthy aging. Neuroimage. 2016; 134: 514–21. DOI: 10.1016/j.neuroimage.2016.04.032 27090351 PMC4912897

[40] Huo X, Xiang J, Wang Y, Kirtman EG, Kotecha R, Fujiwara H, et al. Gamma oscillations in the primary motor cortex studied with MEG. Brain Dev. 2010; 32(8): 619–24. DOI: 10.1016/j.braindev.2009.09.021 19836911

[41] Jurkiewicz MT, Gaetz WC, Bostan AC, Cheyne D. Post-movement beta rebound is generated in motor cortex: evidence from neuromagnetic recordings. Neuroimage. 2006; 32(3): 1281–9. DOI: 10.1016/j.neuroimage.2006.06.005 16863693

[42] Crone NE, Miglioretti DL, Gordon B, Lesser RP. Functional mapping of human sensorimotor cortex with electrocorticographic spectral analysis. II. Event-related synchronization in the gamma band. Brain. 1998; 121(12): 2301–15. DOI: 10.1093/brain/121.12.2301 9874481

[43] Seeber M, Scherer R, Muller-Putz GR. EEG Oscillations Are Modulated in Different Behavior-Related Networks during Rhythmic Finger Movements. J Neurosci. 2016; 36(46): 11671–81. DOI: 10.1523/JNEUROSCI.1739-16.2016 27852775 PMC6705645

[44] Cross KA, Malekmohammadi M, Woo Choi J, Pouratian N. Movement-related changes in pallidocortical synchrony differentiate action execution and observation in humans. Clin Neurophysiol. 2021. DOI: 10.1101/2020.05.27.117416 PMC8286311 33980469

[45] Yanagisawa T, Yamashita O, Hirata M, Kishima H, Saitoh Y, Goto T, et al. Regulation of motor representation by phase-amplitude coupling in the sensorimotor cortex. J Neurosci. 2012; 32(44): 15467–75. DOI: 10.1523/JNEUROSCI.2929-12.2012 23115184 PMC6621589

[46] Salmelin R, Hamalainen M, Kajola M, Hari R. Functional segregation of movement-related rhythmic activity in the human brain. Neuroimage. 1995; 2(4): 237–43. DOI: 10.1006/nimg.1995.1031 9343608

[47] Cassim F, Monaca C, Szurhaj W, Bourriez JL, Defebvre L, Derambure P, et al. Does post-movement beta synchronization reflect an idling motor cortex? Neuroreport. 2001; 12(17): 3859–63. DOI: 10.1097/00001756-200112040-00051 11726809

[48] Hall SD, Barnes GR, Furlong PL, Seri S, Hillebrand A. Neuronal network pharmacodynamics of GABAergic modulation in the human cortex determined using pharmaco-magnetoencephalography. Hum Brain Mapp. 2010; 31(4): 581–94. DOI: 10.1002/hbm.20889 19937723 PMC3179593

[49] Baumgarten TJ, Oeltzschner G, Hoogenboom N, Wittsack HJ, Schnitzler A, Lange J. Beta Peak Frequencies at Rest Correlate with Endogenous GABA+/Cr Concentrations in Sensorimotor Cortex Areas. PLoS One. 2016; 11(6): e0156829. DOI: 10.1371/journal.pone.0156829 27258089 PMC4892568

[50] Baker MR, Baker SN. The effect of diazepam on motor cortical oscillations and corticomuscular coherence studied in man. J Physiol. 2003; 546(Pt 3): 931–42. DOI: 10.1113/jphysiol.2002.029553 12563016 PMC2342588

[51] Cheng CH, Tsai SY, Liu CY, Niddam DM. Automatic inhibitory function in the human somatosensory and motor cortices: An MEG-MRS study. Sci Rep. 2017; 7(1): 4234. DOI: 10.1038/s41598-017-04564-1 28652623 PMC5484662

[52] Nutt D, Wilson S, Lingford-Hughes A, Myers J, Papadopoulos A, Muthukumaraswamy S. Differences between magnetoencephalographic (MEG) spectral profiles of drugs acting on GABA at synaptic and extrasynaptic sites: a study in healthy volunteers. Neuropharmacology. 2015; 88: 155–63. DOI: 10.1016/j.neuropharm.2014.08.017 25195191

[53] Pechadre JC, Beudin P, Trolese JF, Gabet JY, Eschalier A. A comparison of the electroencephalographic spectral modifications induced by diazepam and by hydroxyzine. J Int Med Res. 1993; 21(5): 234–42. DOI: 10.1177/030006059302100502 8112481

[54] Jensen O, Goel P, Kopell N, Pohja M, Hari R, Ermentrout B. On the human sensorimotor-cortex beta rhythm: sources and modeling. Neuroimage. 2005; 26(2): 347–55. DOI: 10.1016/j.neuroimage.2005.02.008 15907295

[55] Muthukumaraswamy SD, Edden RA, Jones DK, Swettenham JB, Singh KD. Resting GABA concentration predicts peak gamma frequency and fMRI amplitude in response to visual stimulation in humans. Proc Natl Acad Sci U S A. 2009; 106(20): 8356–61. DOI: 10.1073/pnas.0900728106 19416820 PMC2688873

[56] Jahanshahi M, Rothwell JC. Inhibitory dysfunction contributes to some of the motor and non-motor symptoms of movement disorders and psychiatric disorders. Philos Trans R Soc Lond B Biol Sci. 2017; 372(1718). DOI: 10.1098/rstb.2016.0198 PMC5332857 28242732

[57] Gironell A. The GABA Hypothesis in Essential Tremor: Lights and Shadows. Tremor Other Hyperkinet Mov (N Y). 2014; 4: 254. DOI: 10.5334/tohm.229 25120944 PMC4108714

[58] Berman BD, Pollard RT, Shelton E, Karki R, Smith-Jones PM, Miao Y. GABAA Receptor Availability Changes Underlie Symptoms in Isolated Cervical Dystonia. Front Neurol. 2018; 9: 188. DOI: 10.3389/fneur.2018.00188 29670567 PMC5893646

[59] Groth CL, Brown M, Honce JM, Shelton E, Sillau SH, Berman BD. Cervical Dystonia Is Associated With Aberrant Inhibitory Signaling Within the Thalamus. Front Neurol. 2020; 11: 575879. DOI: 10.3389/fneur.2020.575879 33633655 PMC7900407

[60] Tapper S, Goransson N, Lundberg P, Tisell A, Zsigmond P. A pilot study of essential tremor: cerebellar GABA+/Glx ratio is correlated with tremor severity. Cerebellum Ataxias. 2020; 7: 8. DOI: 10.1186/s40673-020-00116-y 32607248 PMC7318770

[61] Garibotto V, Romito LM, Elia AE, Soliveri P, Panzacchi A, Carpinelli A, et al. In vivo evidence for GABA(A) receptor changes in the sensorimotor system in primary dystonia. Mov Disord. 2011; 26(5): 852–7. DOI: 10.1002/mds.23553 21370265

[62] Lopes EF, Roberts BM, Siddorn RE, Clements MA, Cragg SJ. Inhibition of Nigrostriatal Dopamine Release by Striatal GABAA and GABAB Receptors. J Neurosci. 2019; 39(6): 1058–65. DOI: 10.1523/JNEUROSCI.2028-18.2018 30541909 PMC6363932

[63] Huang AR, Mallet L, Rochefort CM, Eguale T, Buckeridge DL, Tamblyn R. Medication-related falls in the elderly: causative factors and preventive strategies. Drugs Aging. 2012; 29(5): 359–76. DOI: 10.2165/11599460-000000000-00000 22550966

[64] O’Brien CP. Benzodiazepine use, abuse, and dependence. J Clin Psychiatry. 2005; (66 Suppl 2): 28–33. 15762817

[65] Premoli I, Bergmann TO, Fecchio M, Rosanova M, Biondi A, Belardinelli P, et al. The impact of GABAergic drugs on TMS-induced brain oscillations in human motor cortex. Neuroimage. 2017; 163: 1–12. DOI: 10.1016/j.neuroimage.2017.09.023 28917695

[66] Ferland MC, Therrien-Blanchet JM, Proulx S, Klees-Themens G, Bacon BA, Dang Vu TT, et al. Transcranial Magnetic Stimulation and H(1)-Magnetic Resonance Spectroscopy Measures of Excitation and Inhibition Following Lorazepam Administration. Neuroscience. 2021; 452: 235–46. DOI: 10.1016/j.neuroscience.2020.11.011 33246064

[67] Spooner RK, Wiesman AI, Proskovec AL, Heinrichs-Graham E, Wilson TW. Rhythmic Spontaneous Activity Mediates the Age-Related Decline in Somatosensory Function. Cereb Cortex. 2019; 29(2): 680–8. DOI: 10.1093/cercor/bhx349 29342238 PMC6319169

[68] Rossiter HE, Davis EM, Clark EV, Boudrias MH, Ward NS. Beta oscillations reflect changes in motor cortex inhibition in healthy ageing. Neuroimage. 2014; 91: 360–5. DOI: 10.1016/j.neuroimage.2014.01.012 24440529 PMC3988925

[69] Gaetz W, Macdonald M, Cheyne D, Snead OC. Neuromagnetic imaging of movement-related cortical oscillations in children and adults: age predicts post-movement beta rebound. Neuroimage. 2010; 51(2): 792–807. DOI: 10.1016/j.neuroimage.2010.01.077 20116434

[70] Gao F, Edden RA, Li M, Puts NA, Wang G, Liu C, et al. Edited magnetic resonance spectroscopy detects an age-related decline in brain GABA levels. Neuroimage. 2013; 78: 75–82. DOI: 10.1016/j.neuroimage.2013.04.012 23587685 PMC3716005

[71] Cuypers K, Verstraelen S, Maes C, Hermans L, Hehl M, Heise KF, et al. Task-related measures of short-interval intracortical inhibition and GABA levels in healthy young and older adults: A multimodal TMS-MRS study. Neuroimage. 2020; 208: 116470. DOI: 10.1016/j.neuroimage.2019.116470 31863914 PMC9652063

[72] Hermans L, Leunissen I, Pauwels L, Cuypers K, Peeters R, Puts NAJ, et al. Brain GABA Levels Are Associated with Inhibitory Control Deficits in Older Adults. J Neurosci. 2018; 38(36): 7844–51. DOI: 10.1523/JNEUROSCI.0760-18.2018 30064995 PMC6125814

[73] Hermans L, Levin O, Maes C, van Ruitenbeek P, Heise KF, Edden RAE, et al. GABA levels and measures of intracortical and interhemispheric excitability in healthy young and older adults: an MRS-TMS study. Neurobiol Aging. 2018; 65: 168–77. DOI: 10.1016/j.neurobiolaging.2018.01.023 29494863

[74] Mooney RA, Cirillo J, Byblow WD. GABA and primary motor cortex inhibition in young and older adults: a multimodal reliability study. J Neurophysiol. 2017; 118(1): 425–33. DOI: 10.1152/jn.00199.2017 28424294 PMC5506262

[75] Gehringer JE, Arpin DJ, Heinrichs-Graham E, Wilson TW, Kurz MJ. Practice modulates motor-related beta oscillations differently in adolescents and adults. J Physiol. 2019; 597(12): 3203–16. DOI: 10.1113/JP277326 31045245 PMC7105901

[76] Stagg CJ, Bestmann S, Constantinescu AO, Moreno LM, Allman C, Mekle R, et al. Relationship between physiological measures of excitability and levels of glutamate and GABA in the human motor cortex. J Physiol. 2011; 589(Pt 23): 5845–55. DOI: 10.1113/jphysiol.2011.216978 22005678 PMC3249054

[77] Guerra A, Asci F, Zampogna A, D’Onofrio V, Berardelli A, Suppa A. The effect of gamma oscillations in boosting primary motor cortex plasticity is greater in young than older adults. Clin Neurophysiol. 2021; 132(6): 1358–66. DOI: 10.1016/j.clinph.2021.01.032 33781703

[78] Nowak M, Hinson E, van Ede F, Pogosyan A, Guerra A, Quinn A, et al. Driving Human Motor Cortical Oscillations Leads to Behaviorally Relevant Changes in Local GABAA Inhibition: A tACS-TMS Study. J Neurosci. 2017; 37(17): 4481–92. DOI: 10.1523/JNEUROSCI.0098-17.2017 28348136 PMC5413186

[79] Zhang Z, Jing Y, Ma Y, Duan D, Li B, Holscher C, et al. Driving GABAergic neurons optogenetically improves learning, reduces amyloid load and enhances autophagy in a mouse model of Alzheimer’s disease. Biochem Biophys Res Commun. 2020; 525(4): 928–35. DOI: 10.1016/j.bbrc.2020.03.004 32173530

[80] Burianova H, Marstaller L, Rich AN, Williams MA, Savage G, Ryan M, et al. Motor neuroplasticity: A MEG-fMRI study of motor imagery and execution in healthy ageing. Neuropsychologia. 2020; 146: 107539. DOI: 10.1016/j.neuropsychologia.2020.107539 32629033

[81] Murray JB. Effects of valium and librium on human psychomotor and cognitive functions. Genet Psychol Monogr. 1984; 109(2D Half): 167–97. 6329901

[82] Orgs G, Dombrowski JH, Heil M, Jansen-Osmann P. Expertise in dance modulates alpha/beta event-related desynchronization during action observation. Eur J Neurosci. 2008; 27(12): 3380–4. DOI: 10.1111/j.1460-9568.2008.06271.x 18598273

[83] Del Percio C, Infarinato F, Iacoboni M, Marzano N, Soricelli A, Aschieri P, et al. Movement-related desynchronization of alpha rhythms is lower in athletes than non-athletes: a high-resolution EEG study. Clin Neurophysiol. 2010; 121(4): 482–91. DOI: 10.1016/j.clinph.2009.12.004 20097129

[84] Johari K, Behroozmand R. Event-related desynchronization of alpha and beta band neural oscillations predicts speech and limb motor timing deficits in normal aging. Behav Brain Res. 2020; 393: 112763. DOI: 10.1016/j.bbr.2020.112763 32540134

[85] Heinrichs-Graham E, Hoburg JM, Wilson TW. The peak frequency of motor-related gamma oscillations is modulated by response competition. Neuroimage. 2018; 165: 27–34. DOI: 10.1016/j.neuroimage.2017.09.059 28966082 PMC5826720

[86] Tewarie P, Hunt BAE, O’Neill GC, Byrne A, Aquino K, Bauer M, et al. Relationships Between Neuronal Oscillatory Amplitude and Dynamic Functional Connectivity. Cereb Cortex. 2019; 29(6): 2668–81. DOI: 10.1093/cercor/bhy136 29897408

[87] Pakenham DO, Quinn AJ, Fry A, Francis ST, Woolrich MW, Brookes MJ, et al. Post-stimulus beta responses are modulated by task duration. Neuroimage. 2020; 206: 116288. DOI: 10.1016/j.neuroimage.2019.116288 31654762 PMC6985901

[88] Fry A, Mullinger KJ, O’Neill GC, Barratt EL, Morris PG, Bauer M, et al. Modulation of post-movement beta rebound by contraction force and rate of force development. Hum Brain Mapp. 2016; 37(7): 2493–511. DOI: 10.1002/hbm.23189 27061243 PMC4982082

[89] Bostan AC, Dum RP, Strick PL. Functional Anatomy of Basal Ganglia Circuits with the Cerebral Cortex and the Cerebellum. Prog Neurol Surg. 2018; 33: 50–61. DOI: 10.1159/000480748 29332073

[90] Chiken S, Nambu A. High-frequency pallidal stimulation disrupts information flow through the pallidum by GABAergic inhibition. J Neurosci. 2013; 33(6): 2268–80. DOI: 10.1523/JNEUROSCI.4144-11.2013 23392658 PMC6619164

[91] O’Riordan S, Raymond D, Lynch T, Saunders-Pullman R, Bressman SB, Daly L, et al. Age at onset as a factor in determining the phenotype of primary torsion dystonia. Neurology. 2004; 63(8): 1423–6. DOI: 10.1212/01.WNL.0000142035.26034.C2 15505159

[92] Prasad S, Bhalsing KS, Jhunjhunwala K, Lenka A, Binu VS, Pal PK. Phenotypic Variability of Essential Tremor Based on the Age at Onset. Can J Neurol Sci. 2019; 46(2): 192–8. DOI: 10.1017/cjn.2018.384 30688180

[93] Delamarre A, Meissner WG. Epidemiology, environmental risk factors and genetics of Parkinson’s disease. Presse Med. 2017; 46(2 Pt 1): 175–81. DOI: 10.1016/j.lpm.2017.01.001 28189372

[94] Milardi D, Quartarone A, Bramanti L, Anastasi G, Bertino S, Basile GA, et al. The Cortico-Basal Ganglia-Cerebellar Network: Past, Present and Future Perspectives. Front Syst Neurosci. 2019; 13. DOI: 10.3389/fnsys.2019.00061 31736719 PMC6831548

[95] McCarthy MM, Moore-Kochlacs C, Gu X, Boyden ES, Han X, Kopell N. Striatal origin of the pathologic beta oscillations in Parkinson’s disease. Proc Natl Acad Sci U S A. 2011; 108(28): 11620–5. DOI: 10.1073/pnas.1107748108 21697509 PMC3136295

[96] Udupa K, Chen R. The mechanisms of action of deep brain stimulation and ideas for the future development. Prog Neurobiol. 2015; 133: 27–49. DOI: 10.1016/j.pneurobio.2015.08.001 26296674

[97] Brazhnik E, McCoy AJ, Novikov N, Hatch CE, Walters JR. Ventral Medial Thalamic Nucleus Promotes Synchronization of Increased High Beta Oscillatory Activity in the Basal Ganglia-Thalamocortical Network of the Hemiparkinsonian Rat. J Neurosci. 2016; 36(15): 4196–208. DOI: 10.1523/JNEUROSCI.3582-15.2016 27076419 PMC4829645

[98] Prescott IA, Dostrovsky JO, Moro E, Hodaie M, Lozano AM, Hutchison WD. Reduced paired pulse depression in the basal ganglia of dystonia patients. Neurobiol Dis. 2013; 51: 214–21. DOI: 10.1016/j.nbd.2012.11.012 23201208

[99] Luo F, Kim LH, Magown P, Noor MS, Kiss ZHT. Long-Lasting Electrophysiological After-Effects of High-Frequency Stimulation in the Globus Pallidus: Human and Rodent Slice Studies. J Neurosci. 2018; 38(50): 10734–46. DOI: 10.1523/JNEUROSCI.0785-18.2018 30373767 PMC6580660

[100] Wilson TW, Slason E, Asherin R, Kronberg E, Reite ML, Teale PD, et al. An extended motor network generates beta and gamma oscillatory perturbations during development. Brain Cogn. 2010; 73(2): 75–84. DOI: 10.1016/j.bandc.2010.03.001 20418003 PMC2880229

